# Does ICT Usage Have a Positive or Negative Effect on Taiwanese Older Adults’ Emotional Experiences? The Moderating Role of Basic Psychological Needs Satisfaction

**DOI:** 10.3390/jintelligence11030046

**Published:** 2023-02-27

**Authors:** Chih-Chi Liu, Ya-Ling Wang

**Affiliations:** Department of Adult and Continuing Education, National Taiwan Normal University, Taipei 10610, Taiwan

**Keywords:** older adults, ICT usage, emotional experience, communication application, basic psychological needs satisfaction

## Abstract

While some studies have found that older adults’ use of information and communications technology (ICT) contributes to their positive emotions, others have not. According to previous research, basic psychological needs satisfaction may help us explore the relationships between older adults’ ICT usage and their emotional experience. This study aimed to investigate the moderation effect of older adults’ basic psychological needs satisfaction on the relationship between ICT usage and emotional experience using the experience sampling method via the communication application, Line. At the first phase of the study, we surveyed each participant’s age, gender and satisfaction with basic psychological needs, and afterward, each participant needed to complete their current situation each day throughout the 10-day period. A total of 788 daily experiences of 32 participants (Mage = 63.13; SDage = 5.97, ranging from 52 to 75; 81% women) were collected, and hierarchical linear modeling (HLM) was conducted. Results revealed that ICT usage generally enhanced older adults’ positive emotional experience. Those with satisfied competence needs had stable and positive emotional experiences with or without using ICT, while those without could further promote their positive emotional experience by using ICT. Those with satisfied relatedness needs had more positive emotional experiences when using ICT, while those without had similar emotional experiences with or without ICT.

## 1. Introduction

With the increase in human lifespan and the growth of the older population, improving humans’ wellbeing in an aging society is regarded as an increasingly important research topic. Past studies have found that using information and communications technology (ICT) is related to older adults’ daily life (Coelho 2022; Damant et al. 2017; Hargittai et al. 2019). Continued use of ICT by older adults can also boost their technological literacy and interest (Castilla et al. 2018). The motives for using ICT are different for each person, but judging from the results of use, it may lead to different emotional experiences. If older adults use ICT to socialize and connect with family and friends, it may save them from negative emotions such as loneliness or social isolation (Khosravi et al. 2016; Masi et al. 2011). They may also use ICT to make their lives more convenient, such as by searching for information, using online financial services, etc., similar to young people. However, it has also been found that when ICT is excessively involved in the lives of older adults, it can result in feeling negative emotions (Kwak et al. 2014). Moreover, there may be negative consequences for older adults when operating an unfamiliar technology (Caspi et al. 2019). However, some studies have found that ICT intervention may not have any impact on certain groups of older adults (Cid et al. 2020). The literature indicates that the relationship between ICT usage and older adults’ emotions is not entirely positive or negative. Therefore, the current study aimed to explore older adults’ ICT use and their daily emotional experience.

Prior studies have focused on the direct impact of ICT usage on emotion, but other points of concern have not been addressed. Past studies have elucidated that basic psychological needs satisfaction is helpful for individual growth (Deci and Ryan 2000; Ryan and Deci 2000). Research in the past had indicated that individuals have three different basic psychological needs: autonomy, competence, and relatedness (Deci and Ryan 2000; Ryan and Deci 2000). Autonomy refers to people’s choices in determining their actions and behaviors; competence is related to a person’s confidence in their ability to face the environment or the ability to achieve expected results; relatedness is defined as the degree to which people feel connected to other individuals and groups in a social environment. Additionally, it has been discussed that when basic psychological needs are satisfied, the use of ICT for learning will result in a more positive experience (Fang et al. 2019). However, when people’s basic psychological needs are not satisfied, they may experience negative feelings when using ICT (Allen and Anderson 2018). In short, basic psychological needs satisfaction may cause people to have different experiences when using ICT. For this reason, the current research used basic psychological needs satisfaction as a moderator to explore whether older adults’ basic psychological needs moderate the relationship between emotional experience and ICT usage.

### 1.1. The Relationship between Older Adults’ ICT Usage and Emotional Experience

The increase in the number of older adults using mobile applications can bring convenience to their lives. Some studies suggest that using ICT is beneficial for older adults; for example, Cotten et al.’s (2013) study indicated that older adults’ motivation to use the Internet is to connect with relatives and friends, as they often need more intimate relationship support in their daily lives (Glass and Vander Plaats 2013; Strom and Strom 2015). By using these technologies to connect with others, negative emotions can be reduced (Choi et al. 2012; Morris et al. 2014).

However, not all older adults who use ICT have positive emotional experiences. For example, the complex interface of digital technology is an important reason for the reluctance of older adults to use it (Hutto et al. 2015; Jung et al. 2017). In addition, ICT use can induce negative emotions in older adults who do not want to admit that they need ICT for assisted living (Bright and Coventry 2013). Factors including the lack of help or support, physical aging, and difficulty coordinating with their lifestyle can lead to older adults experiencing negative emotions when using ICT (Kim 2012). Hence, the question remains as to whether the use of ICT increases older adults’ positive emotions.

Nevertheless, what is known from past research is that basic psychological needs satisfaction may moderate the relationship between ICT use and emotion (Allen and Anderson 2018; Przybylski et al. 2009; Fang et al. 2019; James et al. 2022; Partala and Kallinen 2012; Pettersson et al. 2021). For example, Przybylski et al. (2009) found that when people’s basic psychological needs are satisfied, they will have a better experience when playing games on ICT; however, when their satisfaction with basic psychological needs is low, they will experience negative emotions such as stress when playing games with ICT. James et al.’s (2022) study indicated that people who are satisfied with their basic psychological needs promote their positive learning emotions when learning with ICT, while people with unsatisfied basic psychological needs are more likely to experience learning anxiety when they use ICT to learn.

From this, it can be inferred that the basic psychological needs satisfaction may be an essential factor moderating the relationship between ICT use and emotional experience. Therefore, the current study intends to use basic psychological needs satisfaction as a moderating variable. The moderation effect of basic psychological needs satisfaction on the relationship between ICT usage and emotional experience is hereinafter discussed.

### 1.2. Moderation Effects of Basic Psychological Needs Satisfaction on the Relationship between ICT Usage and Emotional Experience

Reis et al. (2000) found that personal daily happiness is affected by these three basic psychological needs; hence, basic psychological needs may affect an individual’s emotional experience. The same situation occurs when using ICT (Partala and Kallinen 2012). In recent years, researchers have been investigating older adults’ basic psychological needs satisfaction (Heissel et al. 2019), and studies have adjusted and verified the psychological needs satisfaction scale for older adults (Heissel et al. 2019; Vanhove-Meriaux et al. 2020). This shows that satisfying basic psychological needs is still important to older adults.

According to the definition of the three basic psychological needs and the past research results, the current study considers that basic psychological needs satisfaction will moderate the relationship between ICT usage and emotional experience. The following assumptions assume that the satisfaction of the three basic psychological needs may be a moderator.

#### 1.2.1. Autonomy Needs Satisfaction

Current research states that autonomy needs satisfaction may moderate the association between ICT use and emotional experience for older adults. Pettersson et al.’s (2021) study using an exercise app as a research tool found that when older adults’ autonomy needs were satisfied, they felt that it was very meaningful to use the app. Conversely, when older adults’ autonomy needs were not met, they experienced negative emotions such as anxiety when using ICT (Di Giacomo et al. 2019). Therefore, the current study proposes the following hypothesis:

**H1.**  *When the autonomy needs of older adults are satisfied, they will have a more positive emotional experience when using ICT. Conversely, if their autonomy needs are not satisfied, they will have less positive emotional experiences when using ICT*.

#### 1.2.2. Competence Needs Satisfaction

Older adults’ competence needs satisfaction is important for their emotional experience when using ICT, especially for those whose competence needs are not met. Previous research has indicated that competence needs satisfaction interferes with the experience of older adults using ICT (Terp et al. 2021). In addition, Korlat et al. (2021) found that people whose competence needs are met have better psychological adaptation and learning attitudes when using ICT to learn. However, older adults with unsatisfied competence needs can have a better emotional experience when using ICT with proper guidance (Choudrie et al. 2020). In case of insufficient ability to use ICT, older adults can still experience more positive emotions through the use of ICT with the help of relatives and friends (Seifert 2020). Therefore, we hypothesize that:

**H2.**  *Older adults whose competence needs are satisfied may have positive and stable emotional experiences with or without ICT use, while older adults whose competence needs are not satisfied may have more positive emotional experiences if they are assisted in integrating ICT use into their daily activities*.

#### 1.2.3. Relatedness Needs Satisfaction

Satisfaction or dissatisfaction with relatedness needs moderates the association between ICT use and emotional experience in older adults. Strutt et al.’s (2022) study showed that older adults whose relatedness needs are satisfied experienced more positive emotions when using ICT. Conversely, older adults are more likely to experience negative emotions when using ICT when their relatedness needs are not met (Delello and McWhorter 2016). Therefore, the current study proposes the following hypothesis:

**H3.**  *When the relatedness needs of older adults are satisfied, they will have more positive emotional experiences when using ICT to integrate into their daily activities. Conversely, when the relatedness needs of older adults are not satisfied, using ICT will not affect their daily emotional experience*.

Through the above, we deduced the possible emotional experience of using ICT when the three basic psychological needs of older adults are and are not satisfied. To investigate the daily emotional experiences of older adults in a timelier fashion, we chose to conduct this study using the experience sampling method (ESM) paired with a communication app (Line). The methodological design of the current study will be illustrated below.

### 1.3. Investigating the Daily Experience of Older Adults Using a Communication Application: The Experience Sampling Methodology

ESM refers to a technology that captures transient states in daily natural environments (Soong et al. 2015) and is one of the techniques for repeatedly measuring older adults’ daily experiences. It captures the transient state of the daily natural environment (Soong et al. 2015) by requiring individuals to record and report their time states, such as emotional experiences, behaviors, and activities, when they occur. The data collected when using the ESM method are similar to the data obtained through a diary, but they are more immediate. In addition, the data collected using this method are less dependent on memory. ESM allows us to understand how individuals respond at different times and in different situations, similar to natural observations, but it is not as invasive as direct observations (Hektner et al. 2007). To use the ESM method to collect data more efficiently, the current research used Line, the most popular instant communication app in Taiwan. Previous studies on the use of communication apps by older adults in Taiwan also used Line as the subject of research (Chen 2019; Yu 2020). For the current research, the benefits of choosing this app are as follows. First, this study aimed to explore the relationship between ICT use and emotional experiences of older adults. It is possible to collect more immediate data by using the Line app to perform such investigations. Furthermore, such a study design fits with Pihlainen et al.’s (2021) suggestion that further research should capture behavioral and emotional changes in older adults when using technology.

Second, it is possible to interact with older adults through communication apps to increase their willingness to take the initiative to fill in survey data. The interactive content includes an introduction to ICT safety, sharing of a common sense of life, and related knowledge on the mental health of older adults. This way, older adults feel that this app is not simply used to record their data but has other more practical uses. Third, older adults can use the app to collect points to redeem rewards at the end of the study. Such an operation can prevent ESM research from being considered a burden. While assisting with the research, older adults can continue to participate to win rewards. This greatly increases their motivation to complete daily surveys.

In summary, it is a trend for older adults to use ICT in their daily lives. Past studies have also shown that ICT usage plays a role in the lives of older adults. The current research, therefore, aimed to explore the emotional impact of the instant experience of older adults using ICT in the natural environment through ESM, using Line, the most commonly used communication app by older adults in Taiwan, as a way to collect ESM data. The level of individual differences in basic psychological needs was also added to determine whether this difference has a moderating effect.

## 2. Materials and Methods

### 2.1. Participants

This study recruited 32 older adults in Taiwan through Line and Facebook posts. Participation conditions include (1) having a smartphone, (2) age over fifty and (3) voluntary participation. The average age was 63.13 years (SD = 5.97, ranging from 52 to 75), and 81% were women (i.e., 26 women and 6 men). The current study participants were predominately female. *The composition of such research participants is similar to some past studies on the ICT use behavior of older adults, with more female participants than male participants* (e.g., Bakshi and Bhattacharyya 2021; Mulasso et al. 2019; Pihlainen et al. 2021; Zhang et al. 2019). For example, the study participants of Bakshi and Bhattacharyya (2021) and Mulasso et al. (2019) consisted of 60% women. In addition, women accounted for 70% of the participants in the study by Vroman et al. (2015). Moreover, the proportion of female participants in the above-mentioned studies exceeds 60%. It can be seen that it is not uncommon for women to participate more actively in similar studies. In order to eliminate the sample bias, the current research uses gender as a control variable and statistically controls the possible impact of gender on the current results. All participants were from metropolitan areas of Taiwan. 96.9% of the participants reported that they used ICT on average multiple times per day, and 3.1% said that they used ICT about once per day. This indicates that the vast majority of participants use ICT daily to a high degree. In addition, 18.8% of the participants reported that they had a graduate degree or higher, 28.1% had a college degree, 40.6% had a high school degree, 9.4% had a junior high school degree, and 3.1% chose not to report their academic qualifications. The basic information about the participants is shown in Table 1.

To enhance the motivation to participate in the research, the researcher provided 100 NT dollars and practical merchandise (such as a pedometer or mobile phone holder) to participants as gifts for participating in the research. The gift choice was based on the degree of cooperation of participants within 10 days. The form of rewards given was determined with reference to past studies (Christian et al. 2014; Spence et al. 2014). In addition, to cope with the problems that may be encountered during the 10-day (in May 2019) research period, and to improve the accuracy of the research data, we provided our contact details to the participants. One participant dropped out before finishing the whole study; therefore, 32 participants completed the study’s diary records. Each participant needed to complete their records every day for 10 days. After deleting the missing data, a total of 788 valid records were collected.

### 2.2. Design: Experience Sampling Methodology

The procedure for experience sampling methodology involves different types of evaluation plans for different research purposes in ESM: (a) interval sampling, which means that the evaluation is carried out at a fixed time point; (b) sampling of accompanying signals, which means that the evaluation is usually performed by signal triggers that occur at different time intervals throughout the day; (c) incidental sampling of events, which refers to evaluations triggered by the occurrence of a predetermined event; and (d) any combination of the above (Hektner et al. 2007). Among these methods, we adopted the method of incidental sampling of signals, requiring the participants to report their experience when receiving online reminder messages on Line.

### 2.3. Ethical Considerations

Before the start of the study, all participants submitted their written informed consent, and a comprehensive report was prepared after the study.

### 2.4. Procedures

First, 15 of the participants were invited to our laboratory for a meeting. They were provided with a research plan and a guidebook, which contained all the steps and information they needed. This research design is also in line with what Pihlainen et al. (2021) pointed out, guiding older adults to use ICT will enhance their willingness and confidence. In the current study, this was very important, as an effort was made to avoid older adults dropping out of the study due to lack of confidence or fear of using ICT. Next, we invited 17 other participants who were not invited to the first meeting to participate in a second meeting with the same content. All participants were assured that their responses would remain completely confidential. They were also taught how to use the communication app, Line, to record their daily lives.

Next, according to the ESM, participants received reminder stickers three times per day for 10 days. The three stickers were sent at semi-random times throughout the day (one each in the morning, afternoon, and evening). Participants did not know when they would receive the message. Each sticker was designed to remind participants to complete the record. In this record, we asked them to reflect on their emotional experience or the current situation and their basic psychological needs satisfaction regarding what had just happened by using a sliding scale when they received the reminder sticker.

Finally, participants returned to the laboratory. According to their performance throughout the research process, they were asked to score the diary records of the past 10 days (i.e., the difficulty in recording). This was the end of the experiment.

### 2.5. Experience Level Measures

Each time participants received a message on the Line app, the researcher would ask them to report the current situation, current activity, and emotional experience over the preceding 30 min (Prentice et al. 2020). The messages contained the following: (1) **ICT Usage**: “Did you use ICT (mobile phones, tablets, computers) to assist you in this activity?” (a. yes; b. no). In this study, participants who answered “a. yes” were coded as 1; participants who answered “b. no” were coded as 0 (61% answered Yes). (2) **Emotional Experience**: “How are you currently feeling” (M = 5.29, SD = 1.19; 1 = very negative, 4 = Normal, 7 = very positive). ICT usage was regarded as the independent variable in the current research, and emotional experience was regarded as the dependent variable.

### 2.6. Individual Level Measures

#### 2.6.1. Basic Psychological Needs Satisfaction

Based on the framework of the Basic Psychological Needs theory (Deci and Ryan 2000), the satisfaction of the three basic psychological needs, i.e., autonomy, competence, and relatedness, are very important for individuals. When basic psychological needs are met, the individual’s development, performance, and happiness are considered to be positively affected. After the participants filled in the basic psychological needs scale, we calculated the scores (Max = 7; Min = 1) of the three dimensions and obtained the results (autonomy: M = 5.29, SD = 0.72; competence: M = 5.44, SD = 0.85; relatedness: M = 5.72, SD = 0.8).

#### 2.6.2. Control Variables

We used the gender and age of the participants as control variables in the current study, where men were coded as 1 and women as 0.

### 2.7. Data Analysis

The current study used SPSS version 23.0 and HLM 8.0 as the statistical analysis tools. The statistical methods used include descriptive statistics, correlation analysis, and hierarchical linear modeling. Descriptive statistics were used to calculate the average and standard deviation of each variable to understand the distribution. Correlation analysis was used to test the linear relationships between variables.

To solve the problem of independence of data structure, we analyzed the data using HLM 8.0 with a hierarchical linear modeling (HLM) framework (Raudenbush and Bryk 2002). In this study (a repeated measures situation), HLM is suitable because it can check the transient state changes over time. The HLM method also allowed us to account for the independence of multiple measures for each individual. As a result, the current research regressed the emotional experience using HLM. For each of the outcome models, Level 1 depicted the experience level involving the current condition (i.e., ICT usage) and emotional experience. Level 2 depicted the individual level involving the control variables (i.e., gender and age) and the basic psychological needs satisfaction (i.e., autonomy, competence, and relatedness).

## 3. Results

Table 1 shows the descriptive statistics of the mean and standard deviation and the correlation analysis between the variables. Among them, Level 1 contains 788 samples, and Level 2 contains 32 samples. The following sections aim to discuss the results and the significance of the analysis individually.

### 3.1. Correlation Analysis

In Table 2, the results of the correlation analysis show that ICT usage and emotional experience revealed a positive correlation. This may represent that older adults’ emotional experience becomes more positive when using ICT. It is also likely that older adults who currently have positive emotional experiences tend to use ICT. In addition, it shows a positive correlation between the three basic psychological needs variables. Additionally, a positive correlation between age and autonomy in basic psychological needs emerged, which means that as age increases, older adults’ autonomy also increases. The coefficient of correlation analysis showed that ICT usage had a low correlation with emotional experience (i.e., 0.09). Therefore, it is more appropriate to use the moderation effect to examine the correlation between two variables in different situations in such circumstances.

### 3.2. The Relationship between Older Adults’ ICT Usage and Emotional Experience

Table 3 presents the HLM analysis results. Model A shows the influence of the control variables; Model B shows the relationship between ICT usage and emotional experience; Model C further analyzes whether the basic psychological needs satisfaction had a moderating effect on Model B. As can be observed in Table 2, Model B shows regression analysis predicting emotional experience by using ICT (b = 0.18, *p* < 0.05). This result indicates that when older adults use ICT (such as using communication apps), their emotional experience becomes more positive. Overall, the current research showed that when older adults use ICT, they have more positive emotional experiences.

### 3.3. Moderation Effects of Basic Psychological Needs Satisfaction on ICT Usage and Emotional Experience

As presented in Table 2, Model C shows that autonomy needs did not have a moderating effect on ICT usage and emotional experience (b = 0.003, *p* > 0.05). This indicates that our H1 is not supported.

**Figure 1:**
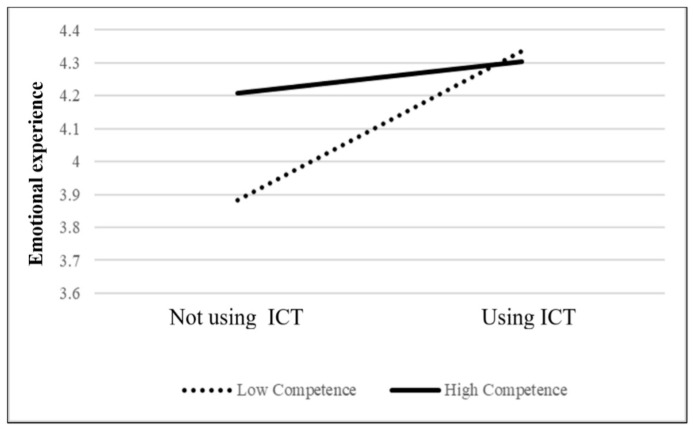
Moderating effect of ICT usage and competence on emotional experience.

**Figure 2:**
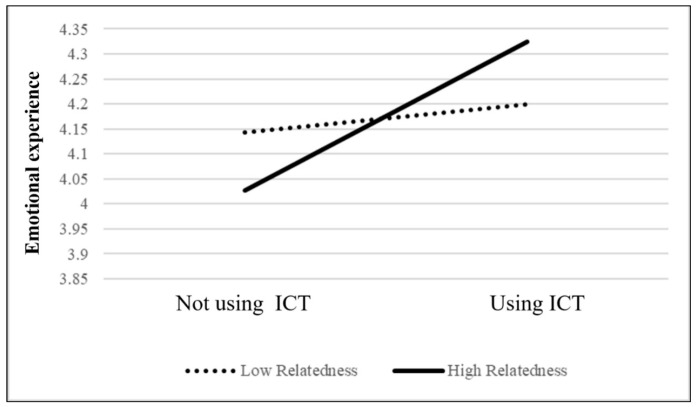
Moderating effect of ICT usage and relatedness on emotional experience.

Moreover, as shown in Table 2 and Figure 1, Model C shows the moderating effect of competence needs satisfaction on ICT usage and emotional experience (b = −0.21, *p* < 0.05). This result indicates that when older adults’ competence needs are satisfied, their emotional experiences tend to be positive. However, when the competence needs of older adults are not satisfied, their emotional experience when using ICT is significantly positive compared to when not using ICT. This supports our H2 that older adults whose competence needs are not satisfied significantly improve their emotional experience using ICT compared with those whose competence needs are satisfied. Finally, as shown in Table 2 and Figure 2, Model C shows a moderating effect of relatedness needs satisfaction on ICT usage and emotional experience (b = 0.21, *p* < 0.05). This supports our H3 that when older adults’ relatedness needs are satisfied, they are more inclined to have more positive emotional experiences when using ICT in their daily lives. This means that when older adults use ICT to connect with relatives and friends, they may have more positive emotional experiences.

## 4. Discussion

The current study shows that the communication app, Line, used on mobile phones is a feasible tool for measuring the daily experience of older adults. First, this data collection method can help obtain more immediate data than paper questionnaires. In addition, past studies have shown that online versions of questionnaires are considered more satisfactory than paper versions (Touvier et al. 2010). Furthermore, older adult participants were more committed to participating in the investigation because we made proper use of the communication app to interact with them and to design a collection of points to make them more willing to use it. Participants in the current study who successfully reported valid data reached a very high percentage. This success can be attributed to the fact that the researchers designed the app based on the needs of older adults and used game design elements, including incentive systems (Plass et al. 2015). The abovementioned data collection results also represent an effective survey tool designed for the current research. This method has seldom been used to measure the emotional experiences of older adults in previous studies. Past studies have also shown that the use of ESM for older adults requires further development (Myin-Germeys et al. 2018). The current research provides an innovative way to use ESM with older adults and can be a reference for future studies. In addition, the current research reveals the daily experiences of older adults and their use of ICT. It is indicated that their experience may be related to their emotional experience. The highlights of this study are discussed below.

### 4.1. Older Adults Have a More Positive Emotional Experience When They Use ICT

It was found that the daily transient experiences of older adults are related to their emotional experiences. When older adults use ICT, they have a more positive emotional experience. This finding is similar to past research showing that using ICT can enhance the positive emotions of older adults (Morris et al. 2014; Tkatch et al. 2021). Moreover, some studies have pointed out that ICT use is positively related to the mental health of older adults (Amoah et al. 2022). Previous research has also pointed out that in the life of older adults, technology can be considered a friend to promote their emotional health (Lee and Kim 2020). In addition, the quarantine policy implemented as a response to COVID-19 has caused older adults to face emotional problems. Past studies have shown that ICT use by older adults can help improve this situation (Llorente-Barroso et al. 2021). However, the association between ICT use in older adults and their feelings still requires more evidence (Wiwatkunupakarn et al. 2022) and careful interpretation of the current study results.

### 4.2. Using ICT Helps Older Adults Whose Competence Needs Are Not Satisfied to Have More Positive Emotional Experiences than Those Whose Competence Needs Are Satisfied

Competence needs satisfaction moderates the relationship between older adults’ ICT usage and emotional experience. The findings of the current research show that when older adults’ competence needs are not satisfied, the use of ICT can further promote their positive emotional experience. Moreover, past studies have shown that when older adults are not familiar with the operation and use of digital technology, they experience negative emotional stress such as anxiety or technological panic (Di Giacomo et al. 2019). However, proper guidance on the use of ICT in older adults can effectively improve their digital self-efficacy (Gatti et al. 2017) and allow them to have better psychological adaptation to ICT use. Therefore, the current findings also point to a meaningful direction for future research to promote positive emotions by assisting older adults whose competence needs are not satisfied to use ICT.

### 4.3. Older Adults Whose Relatedness Needs Are Satisfied Have More Positive Emotional Experiences When Using ICT than Those Whose Relatedness Needs Are Not Satisfied

Relatedness needs satisfaction moderates the relationship between older adults’ ICT usage and emotional experience. Older adults whose relatedness needs are satisfied and use ICT to integrate into their daily activities often have more positive emotional experiences. Based on this finding, we tried to provide an explanation. Research by Cotten et al. (2013) indicated that the main motivation of older adults to use the Internet is to establish contact with relatives and friends. This means that older adults tend to use digital devices to establish connections with friends and family. The results of the current research show that the use of ICT in this way may replace actual social activities with friends and family. For example, in the study of Lüders and Gjevjon (2017), their qualitative interview analysis found that older adults believed that social interaction can increase their willingness to use ICT. It is similar to the abovementioned substitution effect of the use of ICT by older adults for social activities. In addition, the frequency of ICT use among older adults was also found to be negatively associated with their social isolation (Stockwell et al. 2021). Therefore, future research should further explore how to increase the ability of older adults to use ICT, so that they can be more familiar with ICT products while meeting their needs for socializing with relatives and friends and reducing their negative emotions. Intergenerational digital learning has been reported to facilitate communication, solidarity, and social bonding among members of a generation, and the shared use of ICTs has further facilitated intergenerational relationships (Derboven et al. 2012).

Autonomy needs did not moderate the relationship between ICT use and emotional experience. This may be due to the current study design, which encouraged participants to participate voluntarily and did not force them to take our daily survey every time. Instead, we used a designed reward system to encourage them to participate (Zhong and Huang 2016). Older adult participants willingly used our designated app. This design resulted in meeting the autonomy needs of the participants (Goh et al. 2017). Therefore, the above-mentioned reasons may have caused the older adult participants in the current study to have small differences in autonomy needs satisfaction, thereby failing to show a moderating effect.

## 5. Conclusions and Limitations

The current findings should be interpreted with caution. We list some limitations and recommendations based on the current research results and hope to provide some suggestions for future research directions.

First, participants were limited by whether the communication software (Line) could be used frequently. This means that participants in the current study may all be active Line app users. Therefore, we suggest that future research may target older adults who are less adept at using ICT to further investigate the relationship between ICT usage and their daily emotional experience. Alternatively, qualitative methods can be used to explore the associations between older adults’ ICT use and their emotions in-depth, to obtain generalized findings.

Second, the current study participants comprise a high proportion of women. Even though the current findings are statistically controlled for the potential influence of gender, the current results should be interpreted cautiously. In the future, male older adults should be more actively promoted to participate in related research, so as to make a more comprehensive interpretation of older adults with different demographic characteristics.

Furthermore, findings from the current study indicate that older adults whose competence needs are not satisfied or whose relatedness needs are satisfied appear to be the groups recommended for ICT use. Future research may design specific ICT use programs for those with the above characteristics to maximize the benefits of ICT use by older adults.

## Figures and Tables

**Table 1:** Basic information about the participants of current study.

	N (%)		N (%)
**Gender**		**Age Group**	
Male	6 (18.8%)	50 to 59	9 (28.1%)
Female	26 (81.2%)	60 to 69	19 (59.4%)
		over 70	4 (12.5%)
**Education**			
graduate degree or higher	6 (18.8%)	**Frequency of using ICT**	
college degree	9 (28.1%)	many times per day	31 (91.2%)
high school degree	13 (40.6%)	Once per day	1 (2.9%)
junior high school degree	3 (9.4%)	every two to three days	0 (0%)
not reported	1 (3.1%)	once a week	0 (0%)
		barely used	0 (0%)

**Table 2:** Descriptive statistics and correlation analysis.

	Mean	*SD*	MIN	MAX	Correlation Analysis				
**Level 1** (*N* = 788)					1	2			
1. ICT usage	0.61	0.49	0	1	-				
2. Emotional experience	4.2	0.98	1	7	0.09 *	-			
**Level 2** (*N* = 32)					3	4	5	6	7
3. Gender	0.19	0.40	0	1	-				
4. Age	63.13	5.97	52	75	0.26	-			
5. Autonomy	5.29	0.72	3.86	6.71	0.11	0.35 *	-		
6. Competence	5.44	0.85	3.83	7	−0.09	0.15	0.46 **	-	
7. Relatedness	5.72	0.80	4.25	7	−0.22	0.07	0.51 **	0.65 **	-

* *p* < 0.05, ** *p* < 0.01.

**Table 3:** Hierarchal linear modeling analysis of using ICT to predict emotional experience.

	Emotional Experience		
**Level 1**	**Model A**	**Model B**	**Model C**
ICT usage (**X1**)		0.18 *	0.16 *
**Level 2**			
Gender (**M1**)	−0.29	−0.22	−0.22
Age (**M2**)	0.05 **	0.04 *	0.04 *
Autonomy (**M3**)		0.16	0.16
Competence (**M4**)		0.14	0.14
Relatedness (**M5**)		0.03	0.03
**Moderating effect of Level 1 and Level 2**			
**X1 × M3**			0.003
**X1 × M4**			−0.21 *
**X1 × M5**			0.21 *

* *p* < 0.05, ** *p* < 0.01. Note: To reduce the table space, each variable is followed by a code (e.g., X1 stands for “ICT usage,” Y1 stands for “emotional experience,” and M1 stands for “gender”). In addition, in the column representing the moderating effect, an “×” symbol is added between the two codes to indicate which two variables have the moderating effect.

## Data Availability

The raw data supporting the conclusions of this article will be made available by the authors, without undue reservation. The data are not publicly available due to restrictions their containing information that could compromise the privacy of research participants.
